# Supplementary material for: Two-week supplementation of Bifidobacterium adolescentis iVS-1 reduces symptoms associated with lactose intolerance in lactose maldigesters
Source: Gut Microbes Rep. 2025 Jun 4;2(1):2508199. doi: 10.1080/29933935.2025.2508199 (PMC12940102; doi:10.1080/29933935.2025.2508199)
Supplement: Supplemental Material [file KGMR_A_2508199_SM4452.docx]

SUPPLEMENTARY FIGURES for

# Two-week supplementation of *Bifidobacterium adolescentis* iVS-1 reduces symptoms associated with lactose intolerance in lactose maldigesters

Monica Ramakrishnan, Tzu-Wen L. Cross, Anna Clapp Organski, Sindusha Mysore Saiprasad, Abigayle M. R. Simpson, Daniel J. Tancredi, Mallory J. Van Haute, Chloe M. Christensen, Zachery T. Lewis, Thomas A. Auchtung, Jens Walter, Robert Hutkins, & Dennis A. Savaiano

**Supplementary Figure 1. β-galactosidase activity of lysed cells.** β-galactosidase activity of cells grown in GOS (green) or lactose (orange) to mid-log phase, then harvested, lysed, and assayed for 5 min. See Supplementary Table 1 for data. Bars extend to the mean and whiskers span the 95% confidence interval.

**Supplementary Figure 2. Gas reduction in milk.** Change in gas produced by four fecal communities containing 5% by volume of 2% milk, relative to communities with no probiotic added. *p < 0.05 by Mann-Whitney-Wilcoxon test. See Supplementary Table 1 for data and p-values. Box plots show the interquartile range (IQR; boxes), median (line), and 1.5 IQR (whiskers).

**
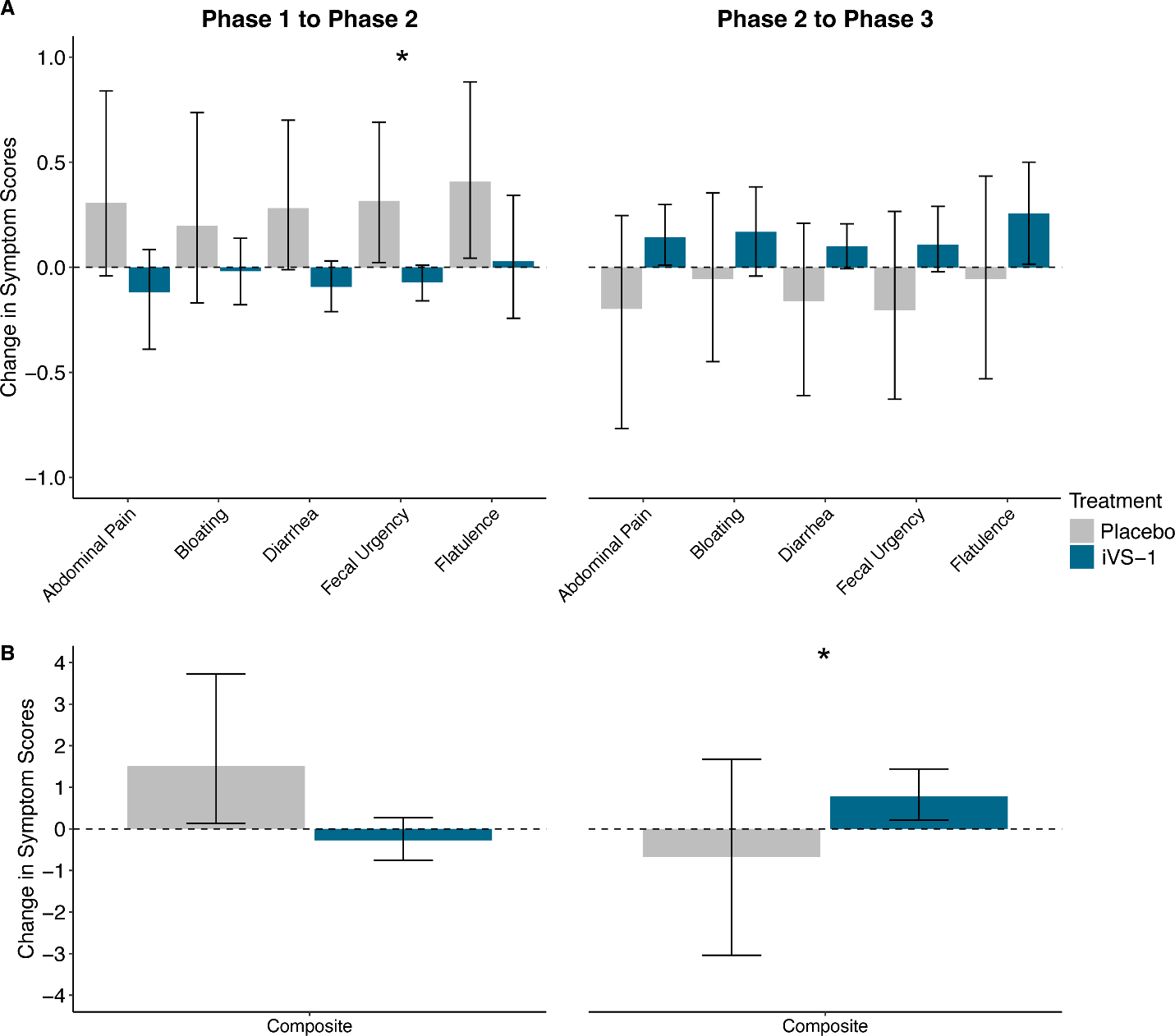
Supplementary Figure 3. Change in patient symptom scores between study phases.** A. Change in mean daily symptom scores for subjects by treatment time period (Phase 1 to Phase 2 and Phase 2 to Phase 3). B. Change in the composite (sum) of all mean daily symptom scores. n = 21 (Phase 1 to 2), 20 (Phase 2 to 3). *p < 0.05 by Mann-Whitney-Wilcoxon test. See Supplementary Table 1 for changes in means and p-values. Bars extend to the mean and whiskers span the 95% confidence interval.

**Supplementary Figure 4. Subject breath hydrogen following lactose consumption.** A. Breath hydrogen over time. Shaded regions represent 95% confidence intervals. B. Cumulative breath hydrogen from seven measurements over six hours. Each subjects' data was normalized for background hydrogen production at time zero. Bars extend to the mean and whiskers span the 95% confidence interval. n = 21 subjects and all comparisons p > 0.05 by Generalized Estimating Equations modeling (A) and Mann-Whitney-Wilcoxon or Wilcoxon signed-rank testing (B). Post-Phase 1 occurred between Phase 1 (before treatment) and Phase 2 (during treatment). Post-Phase 2 occurred between Phase 2 (during treatment) and Phase 3 (after treatment). See Supplementary Table 1 for data and p-values.

**Supplementary Figure 5. Alpha and beta diversity of iVS-1 and placebo groups.** Principal coordinate analysis of unweighted UniFrac distances at post-Phase 1, 2, and 3 (A-C). Alpha diversity assessed by Shannon indices (D), observed features (E), and evenness (F). All p > 0.05 by pairwise PERMANOVA (A-C) and Mann-Whitney-Wilcoxon test (D-F).

**
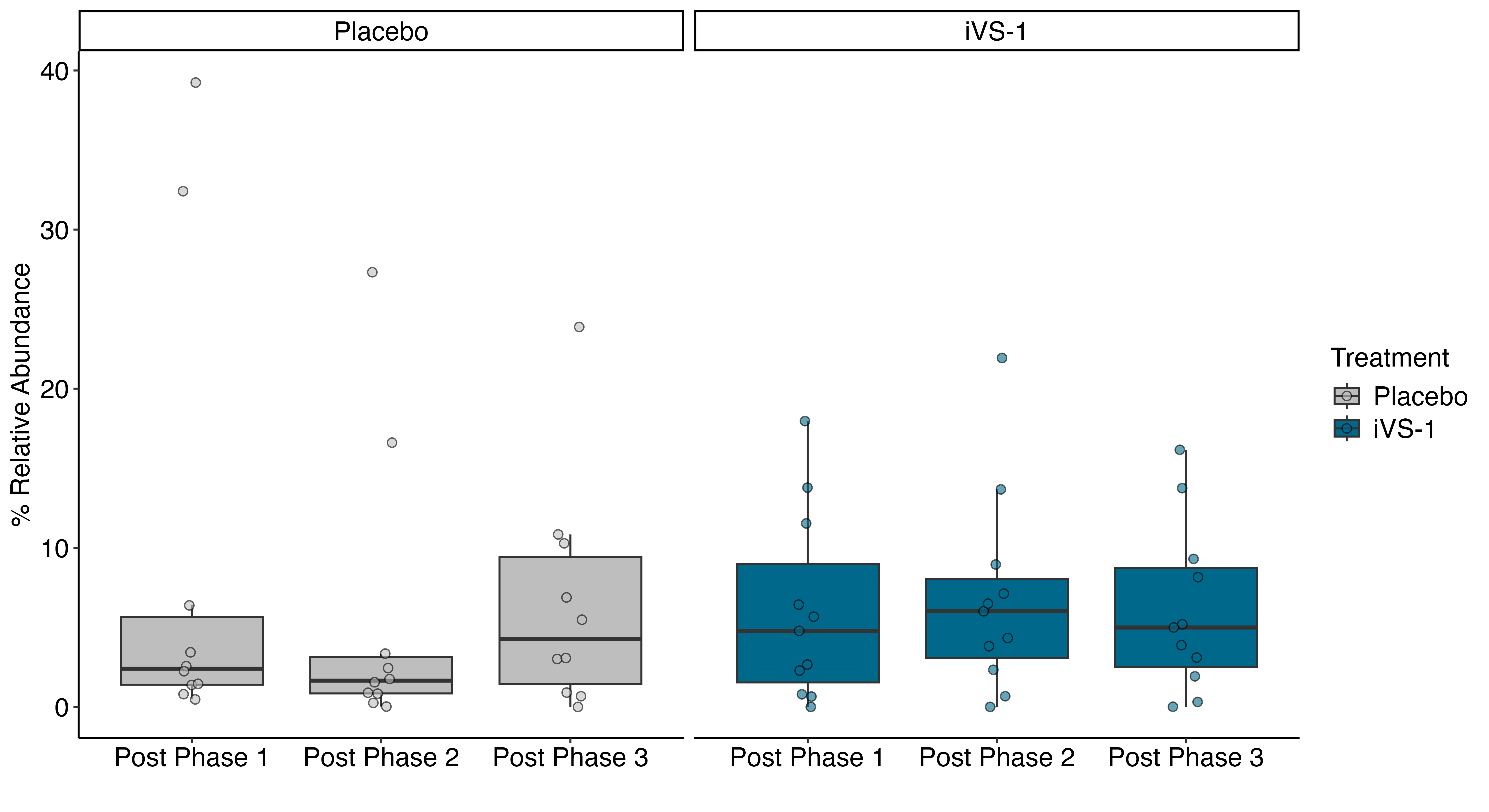
 Supplementary Figure 6. *Bifidobacterium* 16S rRNA gene relative abundance by post Phase.** The relative abundance of *Bifidobacterium* 16S rRNA gene amplicon sequence variants was examined between iVS-1 (n = 11) and placebo (n = 10) groups post Phase 1, 2, and 3, and between phases for each treatment group. All p > 0.05 by Mann-Whitney-Wilcoxon test. See Supplementary Table 1 for data and p-values. Box plots show interquartile range (IQR; boxes), median (line), and 1.5 IQR (whiskers).

**
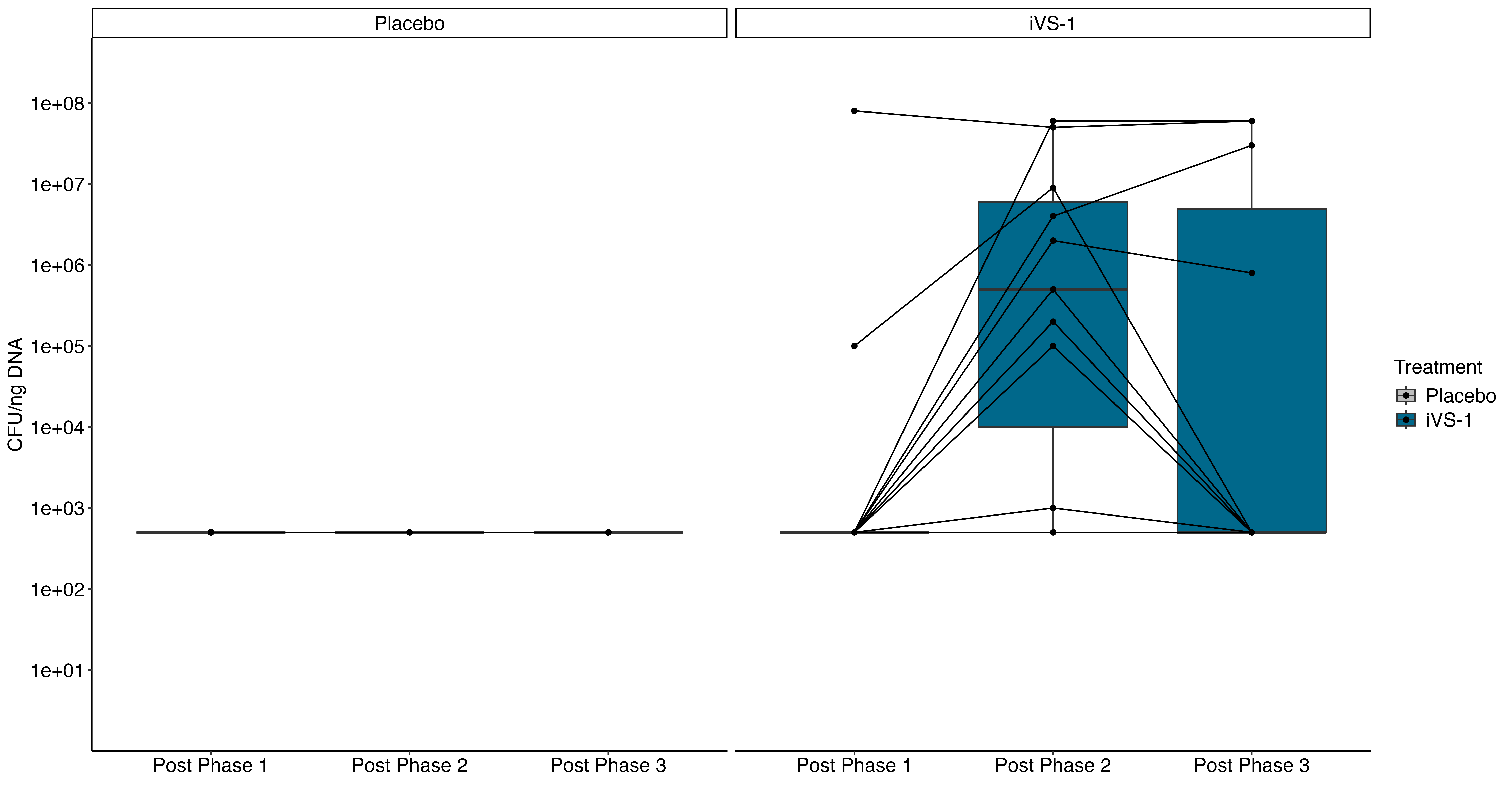
Supplementary Figure 7. Abundance of iVS-1 in fecal samples.** Using qPCR, the abundance of iVS-1 was determined following all three study phases. Points from individual subjects are connected by lines. n = 21. *p = 0.045 and ***p = 0.0005 by Mann-Whitney-Wilcoxon test. See Supplementary Table 1 for means and p-values. Box plots show interquartile range (IQR; boxes), median (line), and 1.5 IQR (whiskers).

**
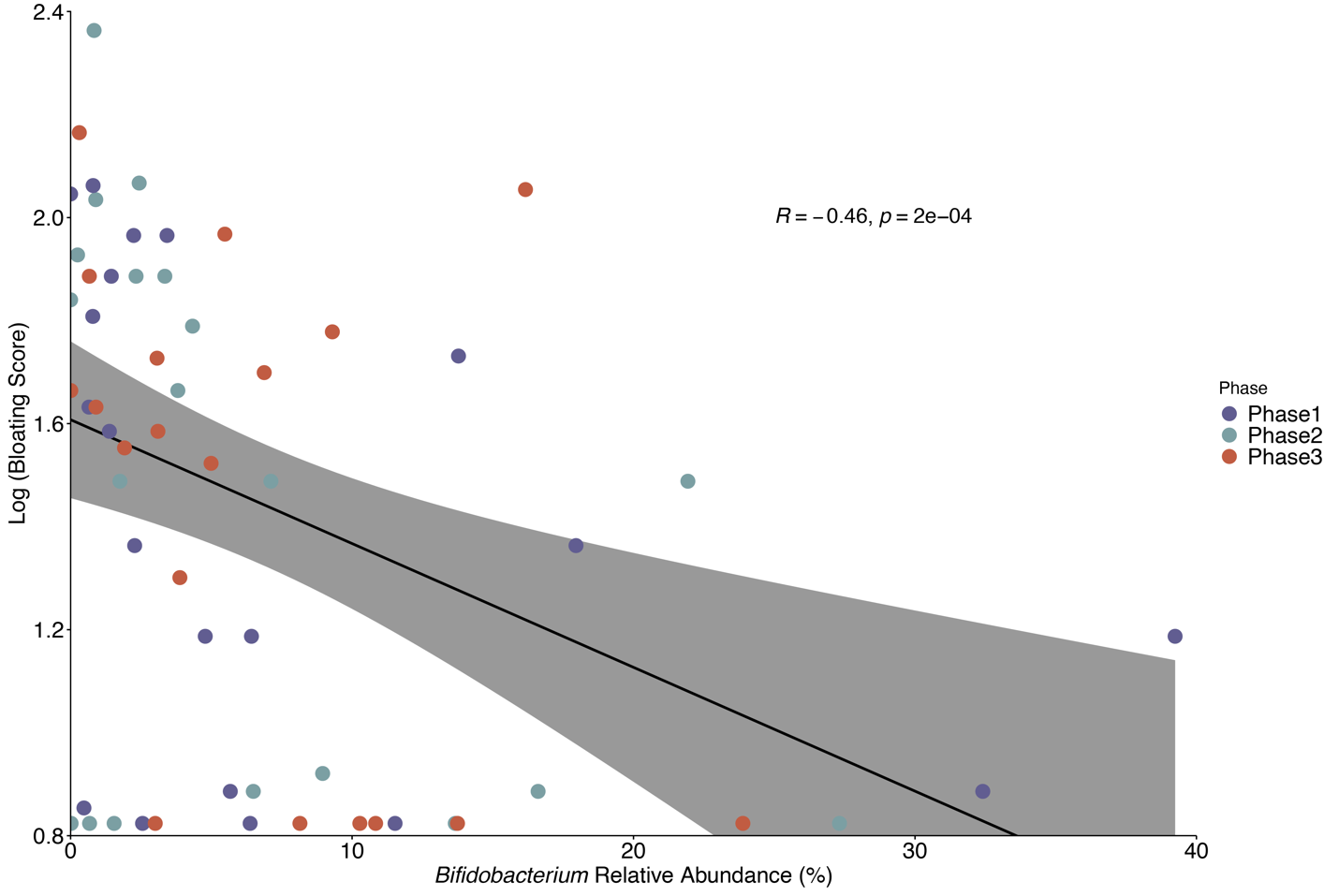
Supplementary Figure 8. Correlation of *Bifidobacterium* relative abundance and bloating scores.** Mean daily bloating scores were multiplied by 100 and log transformed. Subjects with zero symptoms were calculated with the lowest non-zero values to allow logarithmic scaling. Points are mean daily scores for individuals (n = 20) at each phase of the study (colors) matched with the relative abundance of *Bifidobacterium* 16S rRNA genes at the end of that phase. R = -0.46 (p = 0.0002) by Spearman correlation.
