# Supplementary figures and images for: Two-week supplementation of Bifidobacterium adolescentis iVS-1 reduces symptoms associated with lactose intolerance in lactose maldigesters
Source: Gut Microbes Rep. 2025 Jun 4;2(1):2508199. doi: 10.1080/29933935.2025.2508199 (PMC12940102; doi:10.1080/29933935.2025.2508199)

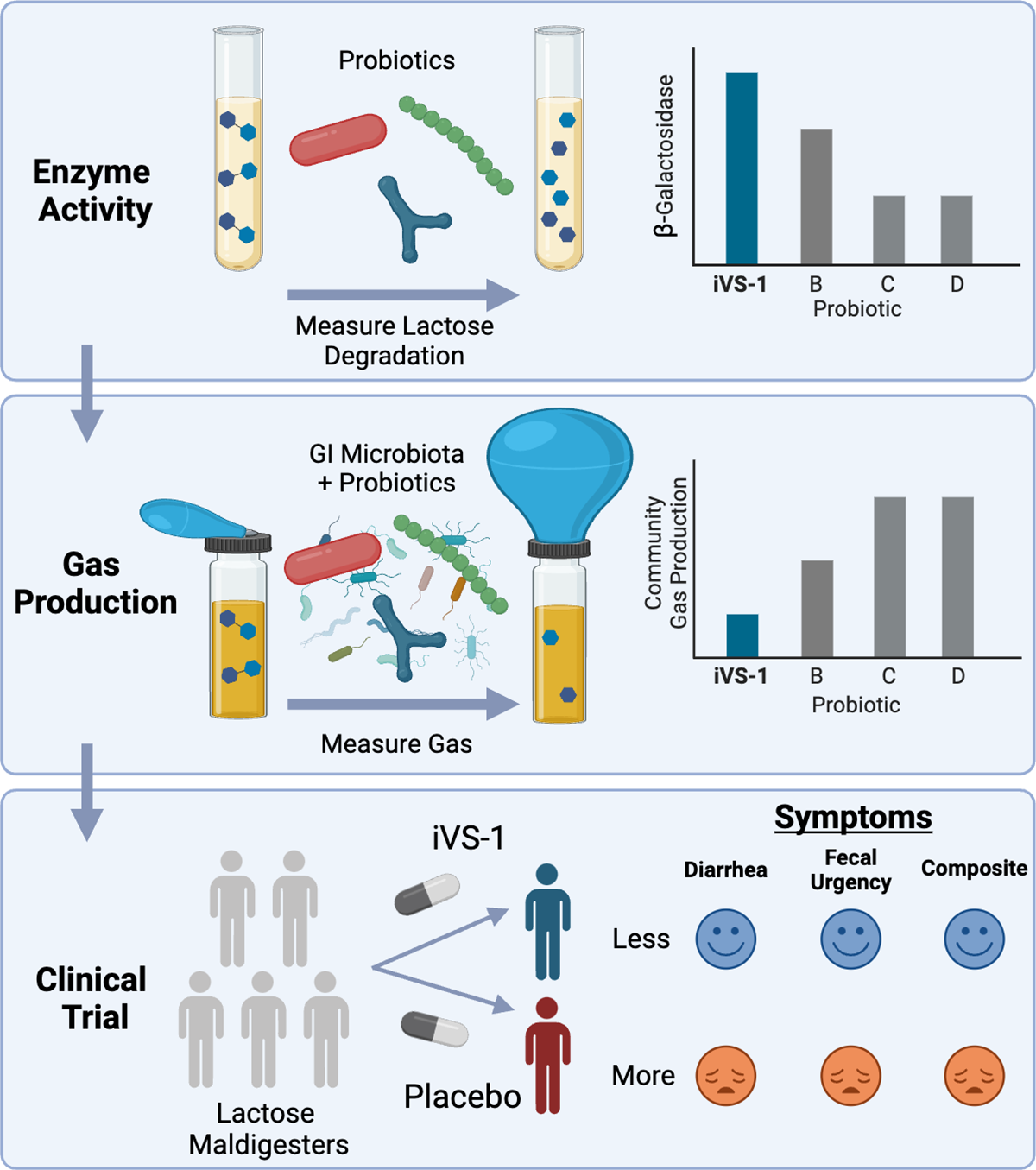

Supplement: Graphical Abstract.png [file KGMR_A_2508199_SM2524.png]
